# Promoter Hypomethylation and miR-145-5p Downregulation- Mediated HDAC11 Overexpression Promotes Sorafenib Resistance and Metastasis of Hepatocellular Carcinoma Cells

**DOI:** 10.3389/fcell.2020.00724

**Published:** 2020-08-12

**Authors:** Wenlong Wang, Bisha Ding, Weiyang Lou, Shengyou Lin

**Affiliations:** ^1^Intensive Care Unit, Hangzhou Hospital of Traditional Chinese Medicine, Hangzhou, China; ^2^Program of Innovative Cancer Therapeutics, Division of Hepatobiliary and Pancreatic Surgery, Department of Surgery, First Affiliated Hospital, College of Medicine, Key Laboratory of Combined Multi-Organ Transplantation, Ministry of Public Health, Key Laboratory of Organ Transplantation, Zhejiang University, Hangzhou, China; ^3^Department of Breast Surgery, First Affiliated Hospital, College of Medicine, Zhejiang University, Zhejiang, China; ^4^Department of Oncology, Hangzhou Hospital of Traditional Chinese Medicine, Hangzhou, China

**Keywords:** hepatocellular carcinoma, sorafenib resistance, metastasis, HDAC11, miR-145-5p, methylation

## Abstract

Sorafenib resistance and tumor metastasis account for poor outcome of hepatocellular carcinoma (HCC). Histone deacetylase 11 (HDAC11) has been reported to exert oncogenic effects in several types of human cancer, but its specific functions and detailed mechanisms in HCC are not fully elucidated. Here we identified HDAC11 as a potential oncogene and promising biomarker in HCC by *in silico* analysis. Histone deacetylase 11 was upregulated in sorafenib-resistant SMMC7721 compared with its parental cell. Knockdown of HDAC11 suppressed proliferation and sorafenib resistance, which may be due to inhibition of drug metabolism cytochrome P450 predicted by gene-set enrichment analysis. Histone deacetylase expression was higher in highly metastatic MHCC97H than lowly metastatic MHCC97L. Downregulation of HDAC11 significantly attenuated the migrated and invaded abilities of HCC cells. Histone deacetylase 11 was directly targeted and suppressed by miR-145-5p. Inhibition of miR-145-5p enhanced sorafenib resistance and metastasis of HCC, and these effects could be attenuated by knockdown of HDAC11. The promoter methylation level of HDAC11 was markedly decreased in HCC tissues compared with normal controls. Administration of 5’-Aza-2’-deoxycytidine, a DNA methyltransferase inhibitor, facilitated HDAC11 expression in HCC cells. Our data indicate a role of miR-145-5p/HDAC11 axis in regulation of sorafenib resistance and metastasis in HCC.

## Introduction

Liver cancer is one of the most leading causes of cancer-associated mortality all over the world (Bray et al., 2018). Hepatocellular carcinoma (HCC) is the most frequent primary liver cancer, accounting for approximately 80% of all cases (McGlynn et al., 2015). Unfortunately, a majority of HCC patients cannot receive surgical resection as they are first diagnosed at advanced stages (Gabutti et al., 2020). The outcome of patients with advanced HCC is still dismal because of limited therapeutic approaches (Lou et al., 2018c). Sorafenib, a multikinase inhibitor, is a clinical first-line systematic treatment agent for advanced HCC approved by the US Food and Drug Administration (Llovet et al., 2008). Acquisition of secondary drug resistance is currently a primary limitation of sorafenib-based chemotherapy (Ding et al., 2018). In addition to sorafenib resistance, vascular invasion and tumor metastasis are also critical factors that lead to poor prognosis and malignant progression of HCC (Lou et al., 2018a). It is extremely imperative to explore the detailed mechanisms responsible for sorafenib resistance, vascular invasion, and tumor metastasis and identify some promising biomarkers for prediction of HCC patients’ prognosis.

Increasing evidences have suggested that epigenetic regulation plays key roles in cancer onset and progression, including drug resistance and metastasis (Yu and Ouyang, 2019; Zhao et al., 2020). Histone deacetylases (HDACs), consisting of 11 members, are a class of critical epigenetic regulators and can be divided into four various subclasses: HDAC I (including HDAC1, HDAC2, HDAC3, HDAC8), HDAC IIa (including HDAC4, HDAC5, HDAC7, HDAC9), HDAC IIb (including HDAC6, HDAC10), and HDAC IV (including HDAC11) (Freese et al., 2019). At present, several HDACs have been reported to be aberrantly expressed and closely associated with tumor malignant progression in HCC. For example, Ler et al. (2015) showed that HDAC1 and HDAC2 could independently predict mortality of HCC using a competing risk regression model; Ji et al. (2019) suggested that HDAC3 deficiency enhanced liver cancer; Fan et al. (2014) discovered that knockdown of HDAC5 suppressed growth of HCC by induction of apoptosis and cell cycle arrest; Gong et al. (2019) suggested that inhibition of HDAC11 could promote apoptosis of human liver cancer. However, the roles and mechanisms of HDAC11 in sorafenib resistance and tumor metastasis of HCC remain not fully elucidated and need to be further studied.

In this study, we first performed a series of analyses for HDACs including expression analysis, receiver operating characteristic (ROC) curve analysis and survival analysis (overall survival and disease free survival) in HCC. Deep investigation revealed that high expression of HDAC11 indicated poor prognosis in HCC with sorafenib treatment and vascular invasion. Next, the functions of HDAC11 in regulation of sorafenib resistance and metastasis were determined in HCC cells. Finally, we explored the detailed molecular mechanisms responsible for HDAC11 overexpression in HCC. Our data in this work may provide key clues for developing effective approaches to overcome sorafenib resistance and tumor metastasis of HCC.

## Materials and Methods

### Expression Analysis

The expression of HDAC family in HCC was determined using The Cancer Genome Atlas (TCGA) and GTEx data by GEPIA^1^, which is a newly developed interactive web server for analyzing the RNA sequencing expression data of 9,736 tumors and 8,587 normal samples (Tang et al., 2017). starBase^2^, a database for exploring microRNA-mRNA interaction maps, was employed to analyze miR-145-5p expression in HCC (Yang et al., 2011; Li et al., 2014). *P* < 0.05 was considered as statistically significant.

### ROC Curve Analysis

The diagnostic values of HDAC family in HCC were assessed by ROC curve analysis using TCGA HCC data. First, the expression values of HDACs were typed into GraphPad Prism software. Then, ROC curve analysis in the “Column Analyses” section was employed to assess their diagnostic values in HCC. *P* < 0.05 was considered as statistically significant.

### Survival Analysis

GEPIA^3^ was utilized to evaluate the prognostic values (overall survival and disease free survival) of HDAC family in HCC. The prognostic values of HDAC11 in sorafenib-treated HCC and HCC with vascular invasion were determined by Kaplan–Meier plotter^4^, which is capable to assess the effect of 54,000 genes on survival in 21 cancer types, including HCC (Nagy et al., 2018). Log-rank *P* < 0.05 was considered as statistically significant.

### Gene-Set Enrichment Analysis

In order to discover the possible action mechanism of HDAC11, gene-set enrichment analysis (GSEA) 3.0 was employed according to the guideline^5^. A total of 371 liver cancer samples were divided into up-expression groups (*n* = 185) and down-expression groups (*n* = 186) in terms of median expression level of HDAC11. c2.cp.kegg.v6.0.symbols.gmt was chosen as the annotated gene sets. *P* < 0.05 was considered as statistically significant.

### Correlation Analysis

The expression correlation between miR-145-5p and HDAC11 was determined using TCGA HCC data by starBase^6^. *P* < 0.05 was considered as statistically significant.

### miRNA Prediction

miRNet^7^, a comprehensive online web server for miRNA-associated studies, was introduced to predict upstream miRNAs that potentially bind to HDAC11, and the predicted binding regions were obtained (Fan et al., 2016; Fan and Xia, 2018).

### Promoter Methylation Analysis

The promoter methylation level of HDAC11 in HCC based on various clinicopathological characteristics was determined using UALCAN^8^, which is a user-friendly and interactive tool for analyzing cancer OMICS data (Chandrashekar et al., 2017).

### Cell Lines and Cell Culture

Four human HCC cell lines, SMMC7721, SMMC7721/SR, MHCC97L, and MHCC97H, and normal liver cell line, QSG7701, were purchased from Shanghai Institute of Biological Science, Chinese Academy of Sciences (Shanghai, China). QSG7701, SMMC7721, and SMMC7721/SR were cultured in Roswell Park Memorial Institute 1640 medium (Gibco, United States), and MHCC97L and MHCC97H were cultured in Dulbecco modified Eagle medium (Gibco, United States) supplemented with 10% fetal bovine serum (FBS) (Gibco, United States) under a humidified atmosphere of 5% CO_2_ at 37°C. To maintain the resistance of SMMC7721/SR, 2,500 ng/mL of sorafenib (Meilunbio, China) was used.

### RNA Isolation and Quantitative Reverse Transcription–Polymerase Chain Reaction Analysis

Total RNA of HCC cells was isolated by RNAiso plus Reagent (Takara, Japan). Subsequently, HDAC11 and miR-145-5p expression was detected by quantitative reverse transcription–polymerase chain reaction as we previously described (Lou et al., 2018b, 2019a). GAPDH and U6 were employed as the internal controls of HDAC11 and miR-145-5p, respectively. The primers used in this study were designed and purchased from RiboBio (Guangzhou, China).

### Protein Extraction and Western Blot Analysis

Total proteins were extracted from HCC cells using RIPA lysis buffer (Beyotime, China). Western blot was used to analyze HDAC11 expression as previously described (Lou et al., 2019b). The primary antibodies of HDAC11 (1:1,000) and GAPDH (1:2,000) were purchased from Abcam, and anti-rabbit peroxidase-conjugated secondary antibody was purchased from Sigma (1:5,000). The band density of HDAC11 was normalized to GAPDH and then quantified using ImageJ software.

### Cell Transfection

siRNA negative control, siRNAs targeting HDAC11 (si-HDAC11#1, si-HDAC11#2), mi-145-5p mimic, inhibitor, and their negative controls, purchased from RiboBio (Guangzhou, China), were transfected into HCC cells using Lipofectamine^TM^ 3000 according to the manufacturer’s instruction.

### CCK-8 Assay for Drug Resistance

Three thousand of SMMC7721 or SMMC7721/SR cells were seeded into 96-well plates and cultured for 12 h, after which cells were transfected as mentioned previously. At 12 h after transfection, the medium was replaced with fresh medium and cultured for 36 h. Next, medium with varied concentrations of sorafenib was added into each well and cultured for 48 h. At the end of culture, 20 μL CCK-8 solution was added into each well and incubated for another 4 h at 37°C. The optical density (OD) value at 450 nm of each well was determined using a microplate reader. The OD values under various drug concentrations were typed into CompuSyn software (version 1.0) and corresponding IC_50_ values were also determined by CompuSyn software (version 1.0).

### Colony Formation Assay

One thousand SMMC7721 or SMMC7721/SR pretransfected cells were seeded into six-well plates and cultured for two 12 days. At the end of culture, these plates were washed with phosphate-buffered saline for twice. Then, these plates were fixed in methanol for 15 min and stained with 0.1% crystal violet solution for another 10 min. Finally, the visible colonies of each well were counted.

### Wound Healing Assay

Wound healing assay was used to assess the migrated abilities of MHCC97L and MHCC97H cells. 50 × 10^4^ pretransfected cells were seeded into six-well plates. When the cells were grown to 100% confluence, “+” wound cross was made in each well using a micropipette tip. Then, photographs were taken immediately, 24 or 48 h after wounding by a microscopy.

### Transwell Invasion Assay

Transwell invasion assay was utilized to evaluate the invaded abilities of MHCC97L and MHCC97H cells. First, Transwell inserts were coated with Matrigel (BD, United States). Then, 0.6 mL medium supplemented with 20% FBS was added into the lower compartment as a chemoattractant. Next, 10 × 10^4^ of MHCC97L and 5 × 10^4^ of MHCC97H cells suspended in 0.2 mL serum-free medium were added into the precoated inserts and cultured for 48 h. At the end of culture, the medium and cells on the upper membrane were carefully removed, and the lower surface were fixed with methanol for 15 min and stained with 0.1% crystal violet solution for another 10 min. Finally, five fields of each well were photographed.

### Dual Luciferase Reporter Assay

Dual luciferase reporter assay was employed to confirm the direct bind of miR-145-5p to HDAC11. First, the 3’ UTR of HDAC11 with or without miR-145-5p binding sites was cloned into psi-CHECK2 vector. Then, these vectors and miR-145-5p mimic or mimic NC were cotransfected into SMMC7721 cells. At 48 h after transfection, the luciferase activity was determined by the Reporter Assay System Kit (Promega, United States) according to the manufacturer’s instruction.

### Administration of DNA Methyltransferase Inhibitor

5’-Aza-2’-deoxycytidine was chosen as a representative DNA methyltransferase inhibitor; 1 × 10^4^ HCC cells were plated into 24-well plates and cultured for 12 h, after which cells were treated with different concentrations of 5’-Aza-2’-deoxycytidine for 72 h. At 72 h later, total RNA of HCC cells was isolated. Histone deacetylase 11 expression levels were detected to assess the role of DNA methyltransferase inhibitor in regulating HDAC11 expression in HCC.

### Statistical Analysis

Statistical analyses of bioinformatics analyses were performed using the online databases as mentioned previously. All experimental data were processed and shown as mean ± SD and analyzed using GraphPad Prism software. Student *t*-test was used to assess differences between two groups. A two-tailed *P* < 0.05 was considered as statistically significant.

## Results

### *In silico* Analyses Identified HDAC11 as a Potential Oncogene in HCC

**FIGURE 1:**
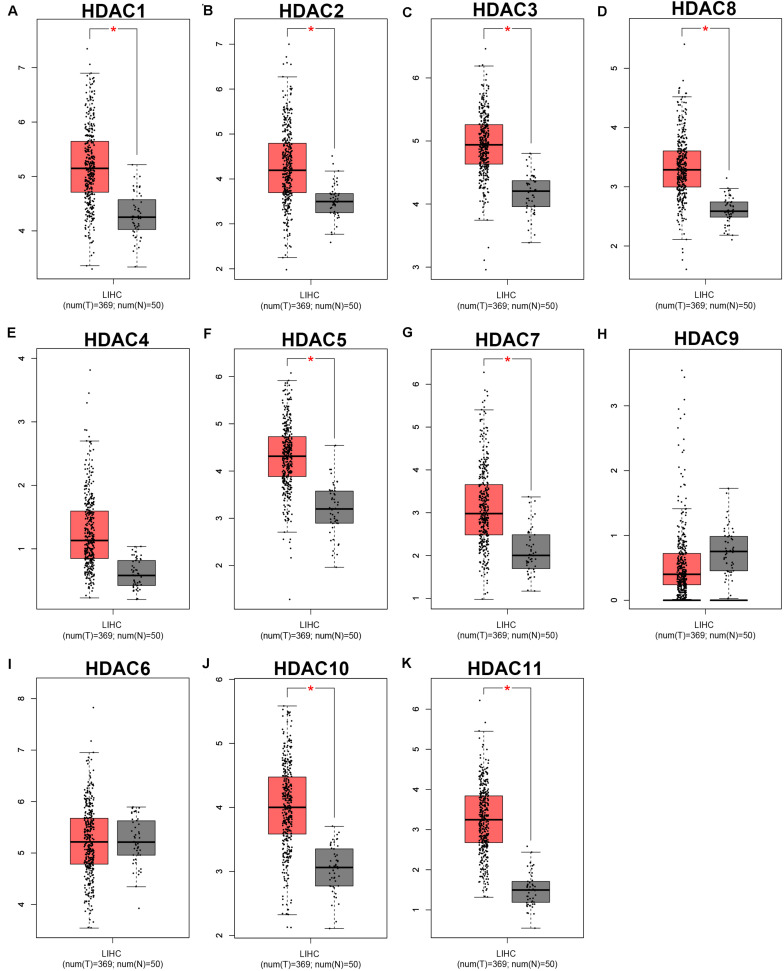
The expression levels of HDAC family members in TCGA HCC tissues compared with TCGA normal liver tissues. **(A)** HDAC1. **(B)** HDAC2. **(C)** HDAC3. **(D)** HDAC8. **(E)** HDAC4. **(F)** HDAC5. **(G)** HDAC7. **(H)** HDAC9. **(I)** HDAC6. **(J)** HDAC10. **(K)** HDAC11. TCGA, The Cancer Genome Atlas. *P < 0.05.

**FIGURE 2:**
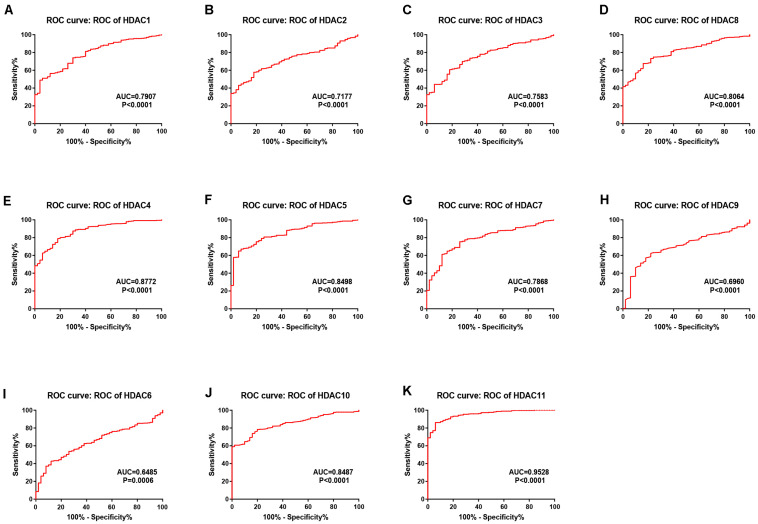
The diagnostic values of HDAC family members in TCGA HCC. **(A)** HDAC1. **(B)** HDAC2. **(C)** HDAC3. **(D)** HDAC8. **(E)** HDAC4. **(F)** HDAC5. **(G)** HDAC7. **(H)** HDAC9. **(I)** HDAC6. **(J)** HDAC10. **(K)** HDAC11.

**FIGURE 3:**
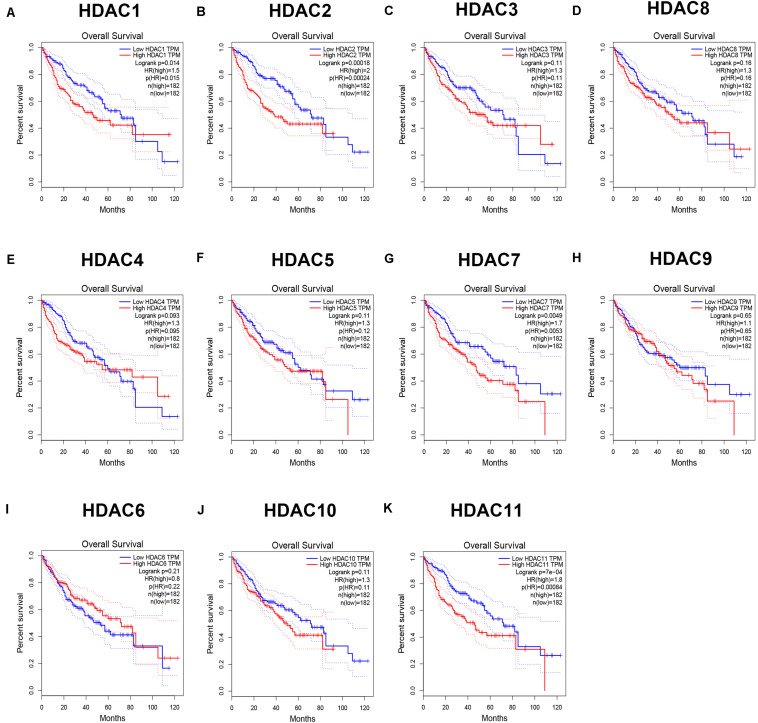
The prognostic values (overall survival) of HDAC family members in TCGA HCC. **(A)** HDAC1. **(B)** HDAC2. **(C)** HDAC3. **(D)** HDAC8. **(E)** HDAC4. **(F)** HDAC5. **(G)** HDAC7. **(H)** HDAC9. **(I)** HDAC6. **(J)** HDAC10. **(K)** HDAC11.

As mentioned previously, the HDAC family has 11 members. First, we analyzed the expression levels of 11 members in HCC using TCGA data (Figure 1) and found that HDAC1, HDAC2, HDAC3, HDAC8, HDAC5, HDAC7, HDAC10, and HDAC11 were significantly upregulated in TCGA HCC tissues compared with TCGA normal controls. Next, we further determined HDACs’ expression in TCGA HCC samples compared with TCGA and GTEx normal controls (Supplementary Figure S1). The result demonstrated that expression of HDAC2, HDAC8, HDAC5, and HDAC11 was markedly increased in HCC. Four members (HDAC2, HDAC8, HDAC5, and HDAC11) were commonly upregulated in two analyses. To ascertain if the 11 members possess significant diagnostic values in HCC, ROC curve analysis was conducted. As presented in Figure 2, all of HDACs could possess the abilities to distinguish HCC samples from normal samples. Subsequently, we also assessed the prognostic values of HDACs in HCC using TCGA data (Figure 3 and Supplementary Figure S2). Patients with HCC with high expression of HDAC1 (Figure 3A), HDAC2 (Figure 3B), HDAC7 (Figure 3G), and HDAC11 (Figure 3K) had unfavorable overall survival. However, only high expression of HDAC11 indicated poor disease free survival in HCC (Supplementary Figure S2K). By combination of expression analysis, ROC curve analysis and survival analysis, we identified HDAC11 as a potential oncogene in HCC (Supplementary Figure S3).

### HDAC11 Depletion Reversed Sorafenib Resistance of HCC *in vitro*

**FIGURE 4:**
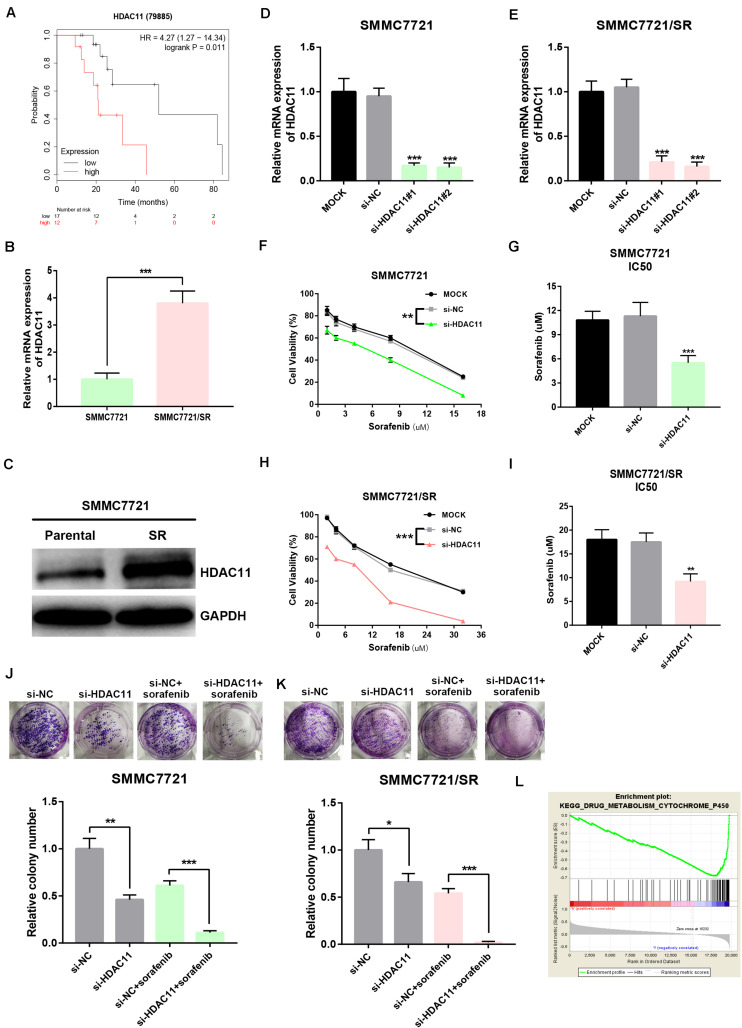
Knockdown of HDAC11 suppressed sorafenib resistance of HCC *in vitro*. **(A)** High expression of HDAC11 indicated poor prognosis in sorafenib-treated HCC from TCGA database. **(B,C)** HDAC11 mRNA and protein were significantly upregulated in SMMC7721/SR compared with SMMC7721. **(D,E)** HDAC11 expression was markedly decreased after treatment of siRNAs targeting HDAC11. **(F,G)** Inhibition of HDAC11 suppressed sorafenib resistance of SMMC7721. **(H,I)** Inhibition of HDAC11 suppressed sorafenib resistance of SMMC7721/SR. **(J,K)** Inhibition of HDAC11 attenuated colony formation of HCC cells. **(L)** GSEA suggested that drug metabolism cytochrome P450 pathway was significantly enriched in HDAC11-low expression group. *P < 0.05, **P < 0.01, ***P < 0.001.

To probe whether HDAC11 is associated with sorafenib resistance of HCC, we first evaluated the prognostic value of HDAC11 in sorafenib-treated HCC samples from TCGA data. As shown in Figure 4A, high expression of HDAC11 indicated poor prognosis in HCC with treatment of sorafenib. Next, we found that HDAC11 mRNA and protein levels were statistically increased in sorafenib resistant SMMC7721 (SMMC7721/SR) when compared with its parental sensitive cell (Figures 4B,C). Considering the high expression of HDAC11, siRNA knockdown method was employed. Histone deacetylase 11 expression was significantly reduced in SMMC7721 and SMMC7721/SR after treatment of siRNAs targeting HDAC11 (Figures 4D–E and Supplementary Figure S4). As shown in Figures 4F–I, silencing HDAC11 suppressed HCC cell proliferation and rendered SMMC7721 and SMMC7721/SR cells more sensitive to sorafenib. Moreover, colony formation assay revealed that inhibition of HDAC11 significantly decreased clonogenic abilities and reversed sorafenib resistance of SMMC7721 (Figure 4J) and SMMC7721/SR (Figure 4K). Notably, the impact of HDAC11 silencing on colony formation seems to be stronger in SMMC7721 as compared to SMMC7721/SR cells. In order to explore the potential action mechanism of HDAC11, we performed GSEA using TCGA HCC data. We found that drug metabolism cytochrome P450 was significantly enriched in HDAC11-low expression group (Figure 4L), implying that HDAC11 might promote sorafenib resistance by suppressing sorafenib metabolism.

### Knockdown of HDAC11 Inhibited Migration and Invasion of HCC *in vitro*

**FIGURE 5:**
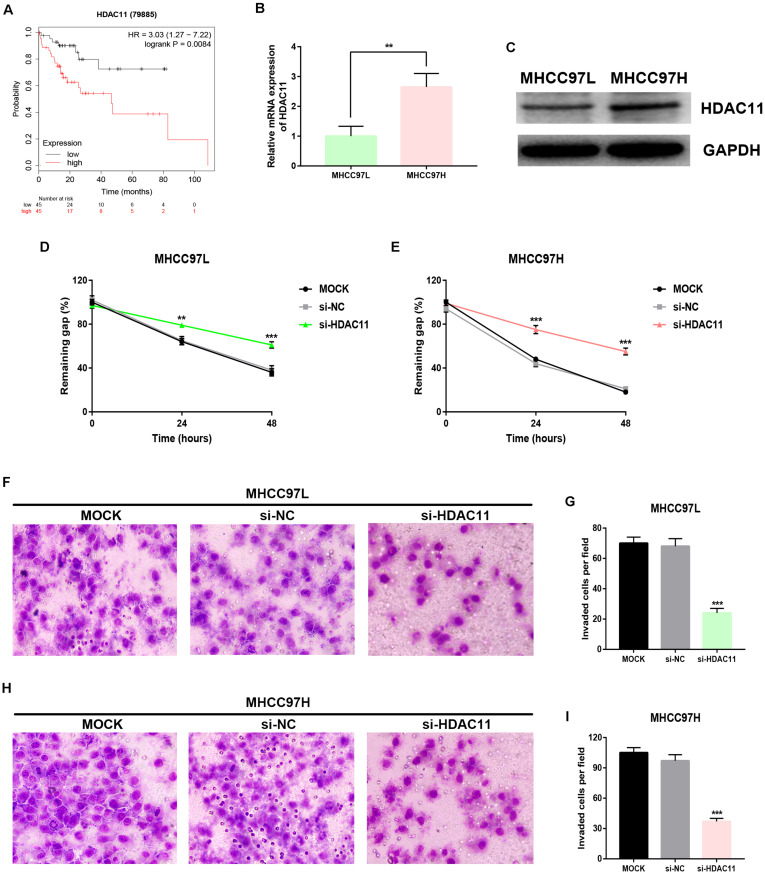
Knockdown of HDAC11 suppressed metastasis of HCC *in vitro*. **(A)** High expression of HDAC11 indicated poor prognosis in HCC with vascular invasion from TCGA database. **(B,C)** HDAC11 mRNA and protein were significantly upregulated in MHCC97H compared with MHCC97L. **(D)** Inhibition of HDAC11 markedly suppressed migration of MHCC97L cell. **(E)** Inhibition of HDAC11 markedly suppressed migration of MHCC97H cell. **(F,G)** Inhibition of HDAC11 markedly suppressed invasion of MHCC97L cell. **(H,I)** Inhibition of HDAC11 markedly suppressed invasion of MHCC97L cell. **P < 0.01, ***P < 0.001.

By analyzing Kaplan–Meier plotter, we showed that high expression of HDAC11 was significantly linked to unfavorable prognosis in HCC with vascular invasion (Figure 5A). Two HCC cell lines, including highly metastatic MHCC97H and lowly metastatic MHCC97L, were employed. Histone deacetylase 11 expression was distinctly increased in MHCC97H compared with MHCC97L (Figures 5B,C). Wound healing assay demonstrated that knockdown of HDAC11 markedly inhibited migration of HCC cells *in vitro* (Figures 5D,E). As presented in Figures 5F–I, downregulation of HDAC11 expression could lead to a significant reduction of *in vitro* invaded abilities of HCC cells. All these findings indicated that knockdown of HDAC11 markedly inhibited migration and invasion of HCC cells.

### miR-145-5p Targeted HDAC11 and Repressed Sorafenib Resistance and Metastasis of HCC *in vitro*

**FIGURE 6:**
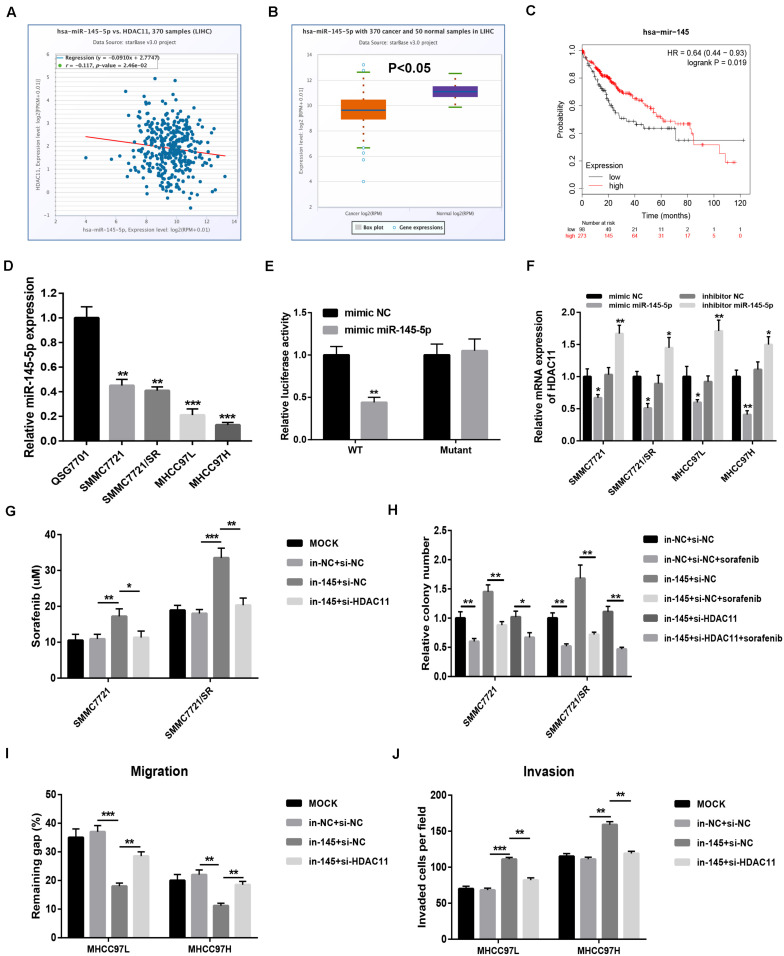
miR-145-5p targeted HDAC11 and repressed sorafenib resistance and metastasis of HCC *in vitro*. **(A)** The expression correlation of HDAC11 and miR-145-5p in HCC determined by starBase. **(B)** miR-145-5p expression was significantly downregulated in HCC tissues compared with normal liver tissues determined by starBase. **(C)** HCC patients with higher expression of miR-145-5p indicated poorer prognosis determined by Kaplan–Meier plotter. **(D)** miR-145-5p expression was markedly decreased in HCC cell lines compared with normal liver cell. **(E)** Dual luciferase reporter assay confirmed the direct bind between miR-145-5p and HDAC11. **(F)** miR-145-5p upregulation significantly decreased HDAC11 expression, whereas miR-145-5p inhibition increased HDAC11 expression in HCC. **(G)** miR-145-5p inhibition increased HCC cell resistance to sorafenib, and this effect can be reversed after knockdown of HDAC11. **(H)** miR-145-5p inhibition promoted HCC cell colony formation, and this effect can be reversed after knockdown of HDAC11. **(I)** miR-145-5p inhibition promoted HCC cell migration, and this effect can be reversed after knockdown of HDAC11. 48 h after wounding. **(J)** miR-145-5p inhibition promoted HCC cell invasion, and this effect can be reversed after knockdown of HDAC11. *P < 0.05, **P < 0.01, ***P < 0.001.

miRNAs are found to negatively regulate gene expression. Using miRNet database, we predicted the potential upstream miRNAs of HDAC11 and found only one binding miRNA, miR-145-5p. The binding sites between miR-145-5p and HDAC11 were shown in Supplementary Figure S5. *In silico* analysis revealed that miR-145-5p was negatively correlated with HDAC11 expression (Figure 6A), significantly downregulated (Figure 6B), and linked to favorable prognosis (Figure 6C) in HCC, indicating that miR-145-5p might be a tumor-suppressive miRNA in HCC. We also confirmed that miR-145-5p expression was markedly decreased in HCC cells compared with normal liver cell (Figure 6D). Dual luciferase reporter assay showed the direct binding of miR-145-5p to HDAC11 (Figure 6E). miR-145-5p could significantly negatively influence HDAC11 expression in HCC cells (Figure 6F and Supplementary Figure S6). Moreover, miR-145-5p inhibition markedly promoted HCC cell sorafenib resistance, migration, and invasion, and knockdown of HDAC11 could statistically attenuate miR-145-5p suppression-mediated enhancement of sorafenib resistance and tumor metastasis in HCC, and miR-145-5p overexpression led to opposite effects (Figures 6G–J and Supplementary Figure S7). Taken together, miR-145-5p targeted HDAC11 and thus reduced HCC cell resistance to sorafenib and suppressed tumor metastasis of HCC *in vitro*.

### Promoter Hypomethylation Level of HDAC11 Was Responsible for HDAC11 Overexpression in HCC

**FIGURE 7:**
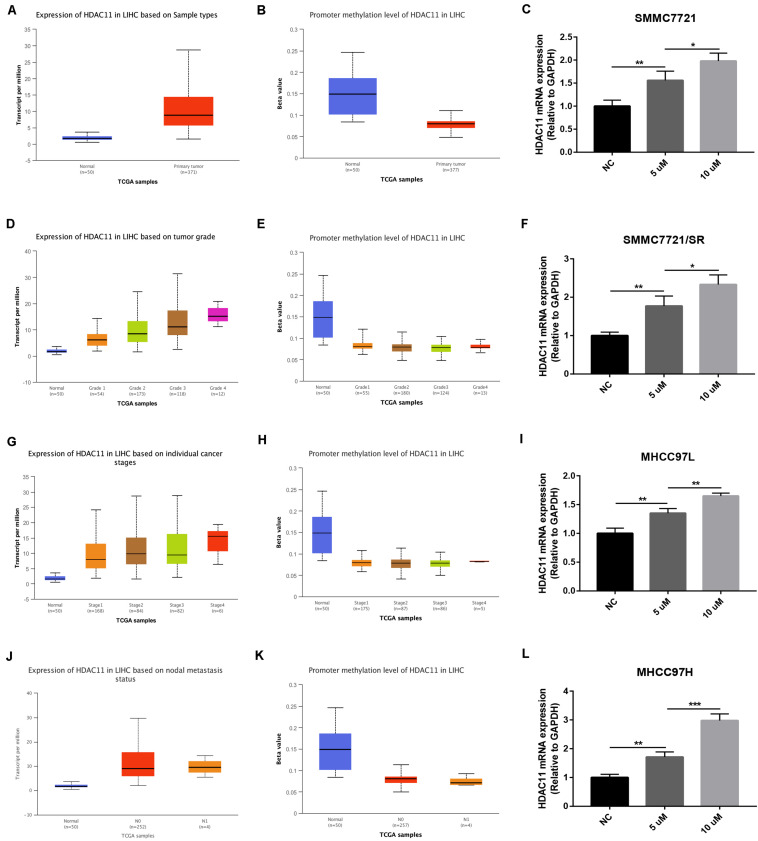
Promoter hypomethylation was responsible for HDAC11 overexpression in HCC. **(A,B)** HDAC11 was upregulated, whereas its promoter methylation level was downregulated in all HCC samples compared with normal controls. **(C,D)** HDAC11 was upregulated, whereas its promoter methylation level was downregulated in HCC based on tumor grade. **(E,F)** HDAC11 was upregulated, whereas its promoter methylation level was downregulated in HCC based on individual cancer stages. **(G,H)** HDAC11 was upregulated, whereas its promoter methylation level was downregulated in HCC based on nodal metastasis status. **(I)** The expression of HDAC11 was significantly increased after treatment of DNA methyltransferase inhibitor 5’-Aza-2’-deoxycytidine in SMMC7721. **(J)** The expression of HDAC11 was significantly increased after treatment of DNA methyltransferase inhibitor 5’-Aza-2’-deoxycytidine in SMMC7721/SR. **(K)** The expression of HDAC11 was significantly increased after treatment of DNA methyltransferase inhibitor 5’-Aza-2’-deoxycytidine in MHCC97L. **(L)** The expression of HDAC11 was significantly increased after treatment of DNA methyltransferase inhibitor 5’-Aza-2’-deoxycytidine in MHCC97H. *P < 0.05, **P < 0.01, ***P < 0.001.

DNA promoter methylation level affects gene expression. Thus, we evaluated the promoter methylation level of HDAC11 in HCC using TCGA data by UALCAN database. As shown in Figures 7A–H, HDAC11 expression was significantly upregulated in HCC, whereas its promoter methylation level was markedly downregulated. These findings suggested that promoter hypomethylation might be involved in the expression regulation of HDAC11. Next, a DNA methyltransferase inhibitor, 5’-Aza-2’-deoxycytidine, was utilized to assess the role of promoter methylation in modulation of HDAC11 expression in HCC. After administration of 5’-Aza-2’-deoxycytidine, HDAC11 expression was significantly upregulated in four HCC cells (Figures 7I–L). Moreover, we found that 5’-Aza-2’-deoxycytidine increased HDAC11 expression in a dose-dependent manner. These results demonstrated that promoter hypomethylation was responsible for HDAC11 overexpression in HCC.

## Discussion

Sorafenib resistance, vascular invasion, and tumor metastasis are several key causes accounting for the high aggressiveness and poor prognosis of HCC. At present, their detailed molecular mechanisms are not totally understood and need to be further investigated. Previous studies have well documented that some HDAC family members act as oncogenes in onset and progression of human cancer, including HCC (Fan et al., 2014; Ler et al., 2015; Ji et al., 2019). However, a systematic research regarding the roles of HDACs in regulation of HCC progression, especially sorafenib resistance and tumor metastasis, remains absent.

By combination of a series of *in silico* analyses, we identified HDAC11 as the most potential member among HDAC family in HCC. Histone deacetylase 11 was significantly upregulated, possessed significant diagnostic value, and negatively correlated with survival time in HCC. Histone deacetylase 11 has been reported to be linked to carcinogenesis of multiple cancer types. For example, Yi et al. (2019) demonstrated that HDAC11 upregulation could suppress basal-like breast cancer cell invasion and metastasis; Wang et al. (2017) showed that HDAC11 inhibited p53 expression in pituitary tumor cells. Moreover, Gong et al. (2019) reported that depletion of HDAC11 promoted liver cancer cell apoptosis. However, the roles of HDAC11 in regulation of sorafenib resistance and metastasis in HCC remain unknown.

Survival analysis revealed that HDAC11 expression was significantly linked to poor prognosis in sorafenib-treated HCC. Following functional experiments demonstrated that knockdown of HDAC11 could reverse sorafenib resistance in HCC cells. By GSEA, the potential action mechanisms of HDAC11 in modulation of sorafenib resistance in HCC were explored. The result suggested that drug metabolism cytochrome P450 pathway was significantly enriched in HDAC11-low expression group, indicating that HDAC11 might promote HCC cell sorafenib resistance by inactivation of drug metabolism cytochrome P450 pathway, which has been confirmed to be involved in drug metabolism in liver (Almazroo et al., 2017; Fuscoe et al., 2020). Moreover, survival analysis also demonstrated that HDAC11 was a promising prognostic biomarker in HCC with vascular invasion. Wounding healing assay and Transwell assay showed the oncogenic roles of HDAC11 in migration and invasion of HCC cells.

Next, we probed the detailed mechanisms responsible for HDAC11 upregulation in HCC. miRNAs, a class of small non-coding RNAs, play key roles in posttranscriptionally regulating of gene expression (Ding et al., 2018; Lou et al., 2018c). miR-145-5p was predicted to potentially bind to HDAC11. *In silico* analysis demonstrated that miR-145-5p was negatively correlated with HDAC11 expression, downregulated in cancer tissues, and indicated poor prognosis in HCC. miR-145-5p has been found to function as a tumor-suppressive miRNA in multiple malignant tumors (Chen et al., 2019, 2020; Zhang et al., 2019). Moreover, miR-145 relevance in HCC has been already reported by Huang et al. (2018). We, for the first time, demonstrated the roles of miR-145-5p in suppressing sorafenib resistance and metastasis and the direct bind of miR-145-5p and HDAC11 in HCC. These findings showed a pivotal role of miR-145-5p/HDAC11 in modulating HCC cell sorafenib resistance and metastasis.

DNA promoter methylation level is also linked to gene transcriptional activity (Jiang et al., 2016; Mohapatra et al., 2020). Data mining revealed a significant reduction of HDAC11 promoter methylation along with overexpression of HDAC11 mRNA in HCC. Furthermore, HDAC11 expression was obviously decreased after treatment of 5’-Aza-2’-deoxycytidine, a DNA methyltransferase inhibitor. Taken together, hypomethylation of HDAC11 promoter might be another mechanism that accounted for HDAC11 overexpression in HCC.

**FIGURE 8:**
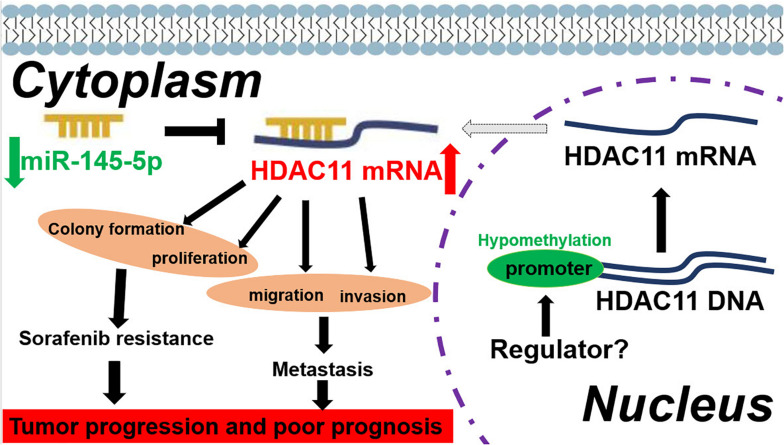
The model of promoter hypomethylation/miR-145-5p/HDAC11 axis in regulation of sorafenib resistance and metastasis of HCC.

Collectively, the current findings elucidated the key roles of HDAC11 in mediating sorafenib resistance and tumor metastasis; miR-145-5p was an upstream regulatory miRNA of HDAC11 in sorafenib resistance and metastasis of HCC; and HDAC11 promoter hypomethylation was also responsible for HDAC11 upregulation in HCC (Figure 8).

However, some limitations presented in our current work: (1) the specific regulators causing promoter hypomethylation need to be further mined; (2) the downstream key targets or pathways (drug metabolism cytochrome P450) of HDAC11 in HCC need to be further investigated; (3) *in vivo* experiments should be performed to further validate the roles of miR-145-5p/HDAC11 axis in sorafenib resistance and metastasis of HCC in the future; and (4) the effect of 5’-Aza-2’-deoxycytidine treatment or HDAC11 methylation dysregulation in sorafenib resistance and metastasis of HCC should be deeply studied.

## Conclusion

Promoter hypomethylation and downregulated miR-145-5p commonly mediated HDAC11 overexpression in HCC; knockdown of HDAC11 could reverse HCC cells resistance to sorafenib and suppress metastasis of HCC cells *in vitro*; miR-145-5p downregulation promoted sorafenib resistance and metastasis *in vitro*, and these effects could be attenuated after inhibition of HDAC11. These findings suggest that miR-145-5p overexpression and HDAC11 depletion may represent two potential approaches to overcome sorafenib resistance and tumor metastasis of HCC in the future.

## Data Availability Statement

The original contributions presented in the study are included in the article/Supplementary Material, further inquiries can be directed to the corresponding author/s.

## Author Contributions

WW, WL, and SL designed this work, performed the experiments, analyzed the data, and drafted the manuscript. BD performed some experiments. WW and SL revised the manuscript. All authors read and approved the final version of the manuscript.

## Conflict of Interest

The authors declare that the research was conducted in the absence of any commercial or financial relationships that could be construed as a potential conflict of interest.

## Footnotes

1 http://gepia.cancer-pku.cn/index.html

2 http://starbase.sysu.edu.cn/index.php

3 http://gepia.cancer-pku.cn/index.html

4 http://kmplot.com/analysis/

5 http://www.broadinstitute.org/gsea/

6 http://starbase.sysu.edu.cn/index.php

7 http://www.mirnet.ca/

8 http://ualcan.path.uab.edu/index.html

## Supplementary Material

FIGURE S1 The expression levels of HDACs family members in TCGA HCC tissues compared with TCGA and GTEx normal liver tissues. **(A)** HDAC1. **(B)** HDAC2. **(C)** HDAC3. **(D)** HDAC8. **(E)** HDAC4. **(F)** HDAC5. **(G)** HDAC7. **(H)** HDAC9. **(I)** HDAC6. **(J)** HDAC10. **(K)** HDAC11. TCGA: The Cancer Genome Atlas. GTEx: Genotype-Tissue Expression. ^∗^*P* < 0.05.

FIGURE S2 The prognostic values (disease free survival) of HDACs family members in TCGA HCC. **(A)** HDAC1. **(B)** HDAC2. **(C)** HDAC3. **(D)** HDAC8. **(E)** HDAC4. **(F)** HDAC5. **(G)** HDAC7. **(H)** HDAC9. **(I)** HDAC6. **(J)** HDAC10. **(K)** HDAC11.

FIGURE S3 The visual flow-process diagram of bioinformatic analyses in this study.

FIGURE S4 The knockdown effect of siRNAs targeting HDAC11 in HCC cells at protein level.

FIGURE S5 The predicted sequences binding regions between wild-type (wt) or mutant (mut) 3′-UTRs of HDAC11 and miR-145-5p.

FIGURE S6 Overexpression of miR-145-5p significantly reduced HDAC11 protein level in SMMC7721 and MHCC97H cells.

FIGURE S7 The roles of miR-145-5p/HDAC11 axis in sorafenib resistance and metastasis of HCC cells. **(A)** miR-145-5p overexpression decreased HCC cells resistance to sorafenib, and this effect can be enhanced after knockdown of HDAC11. **(B)** miR-145-5p overexpression suppressed HCC cell colony formation, and this effect can be enhanced after knockdown of HDAC11. **(C)** miR-145-5p overexpression inhibited HCC cell migration, and this effect can be enhanced after knockdown of HDAC11. 48 h after wounding. **(D)** miR-145-5p overexpression suppressed HCC cell invasion, and this effect can be enhanced after knockdown of HDAC11. ^∗^*P* < 0.05; ^∗∗^*P* < 0.01; ^∗∗∗^*P* < 0.001.

## Abbreviations

FBS: fetal bovine serum
GSEA: gene-set enrichment analysis
HCC: hepatocellular carcinoma
HDAC11: histone deacetylase 11
HDACs: histone deacetylases
OD: optical density
ROC: receiver operating characteristic.
