# Supplementary figures and images for: Promoter Hypomethylation and miR-145-5p Downregulation- Mediated HDAC11 Overexpression Promotes Sorafenib Resistance and Metastasis of Hepatocellular Carcinoma Cells
Source: Front Cell Dev Biol. 2020 Aug 12;8:724. doi: 10.3389/fcell.2020.00724 (PMC7434871; doi:10.3389/fcell.2020.00724)

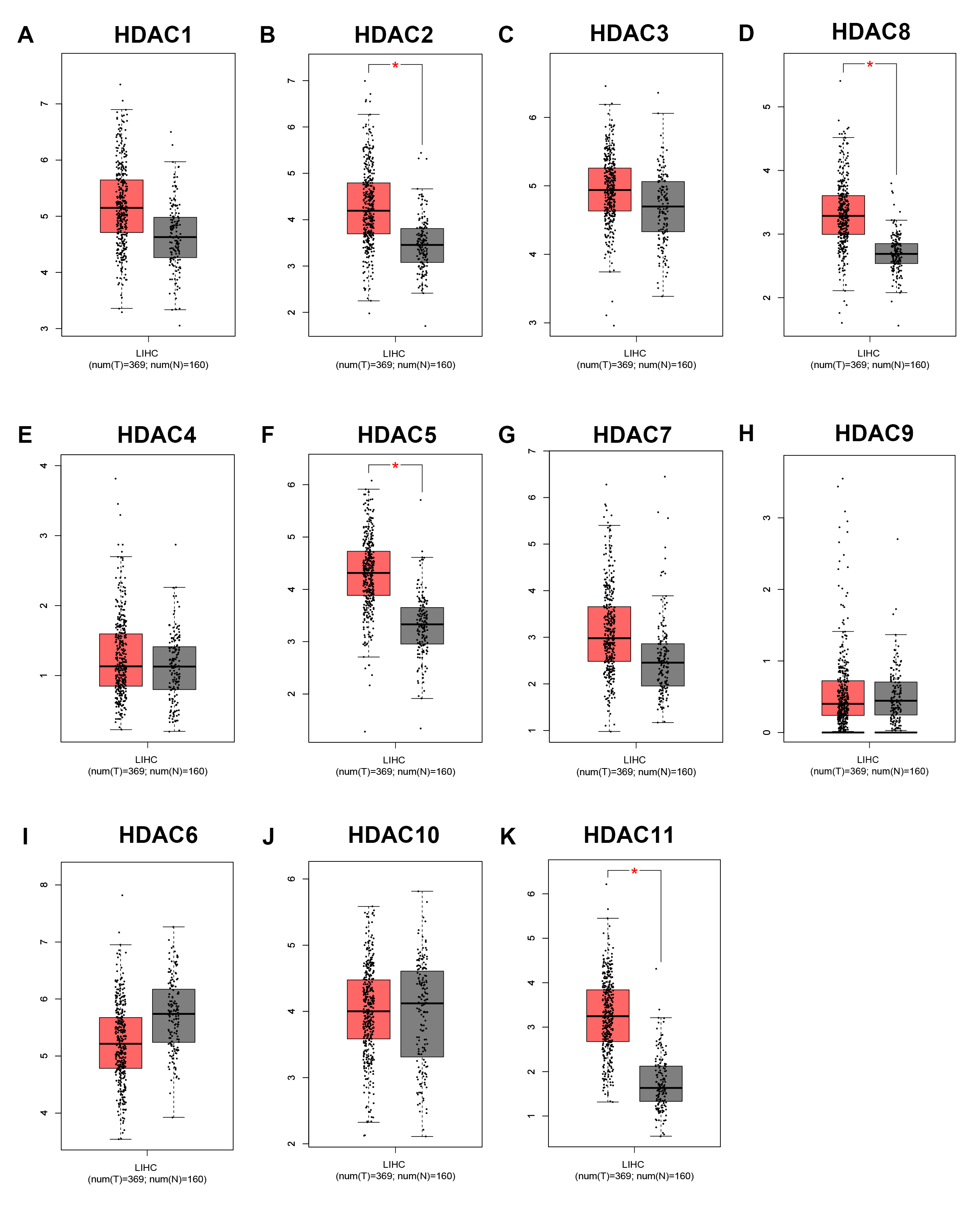

Supplement: FIGURE S1 — The expression levels of HDACs family members in TCGA HCC tissues compared with TCGA and GTEx normal liver tissues. (A) HDAC1. (B) HDAC2. (C) HDAC3. (D) HDAC8. (E) HDAC4. (F) HDAC5. (G) HDAC7. (H) HDAC9. (I) HDAC6. (J) HDAC10. (K) HDAC11. TCGA: The Cancer Genome Atlas. GTEx: Genotype-Tissue Expression. ∗P < 0.05. [file Image_1.jpeg]

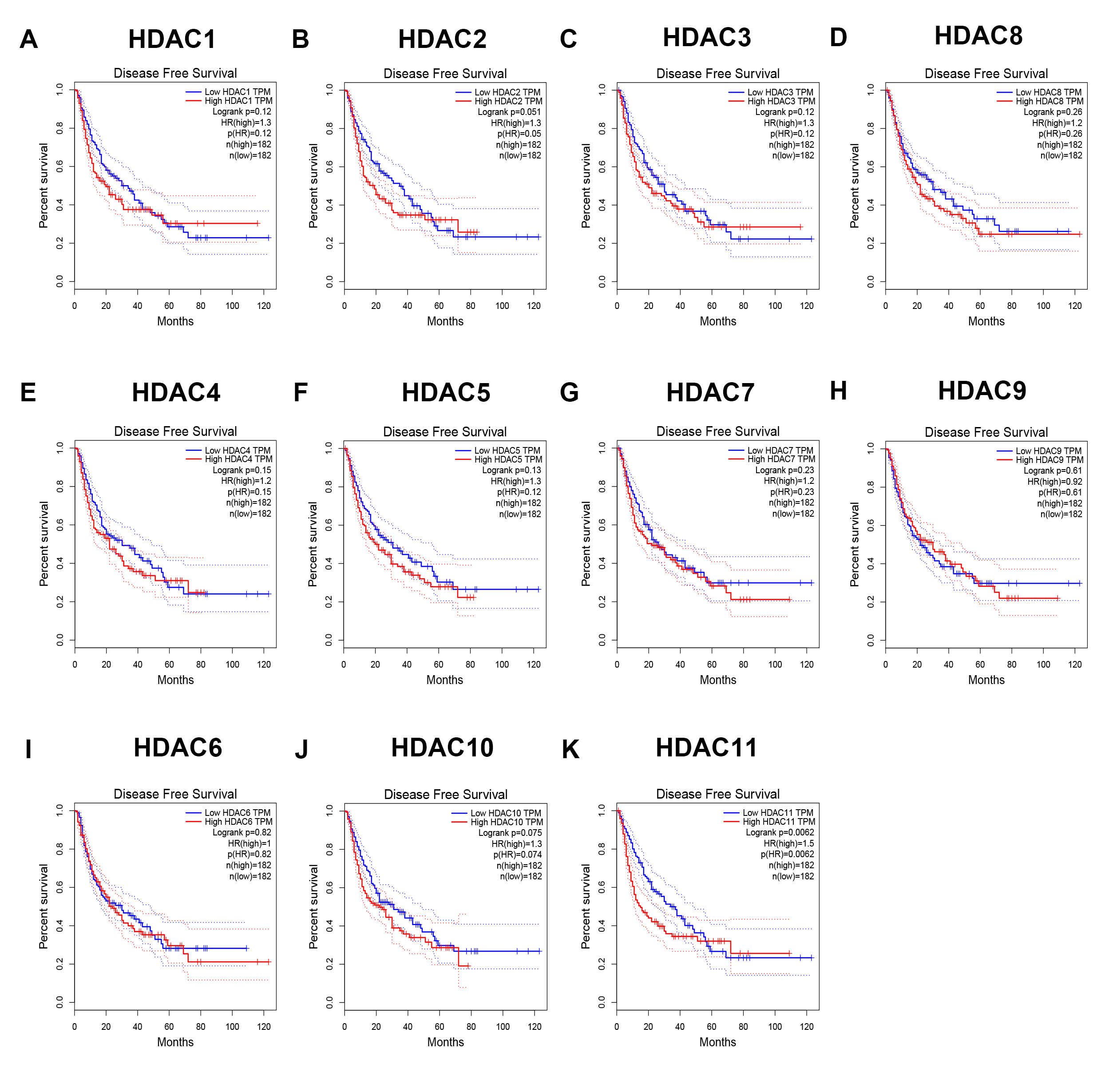

Supplement: FIGURE S2 — The prognostic values (disease free survival) of HDACs family members in TCGA HCC. (A) HDAC1. (B) HDAC2. (C) HDAC3. (D) HDAC8. (E) HDAC4. (F) HDAC5. (G) HDAC7. (H) HDAC9. (I) HDAC6. (J) HDAC10. (K) HDAC11. [file Image_2.jpeg]

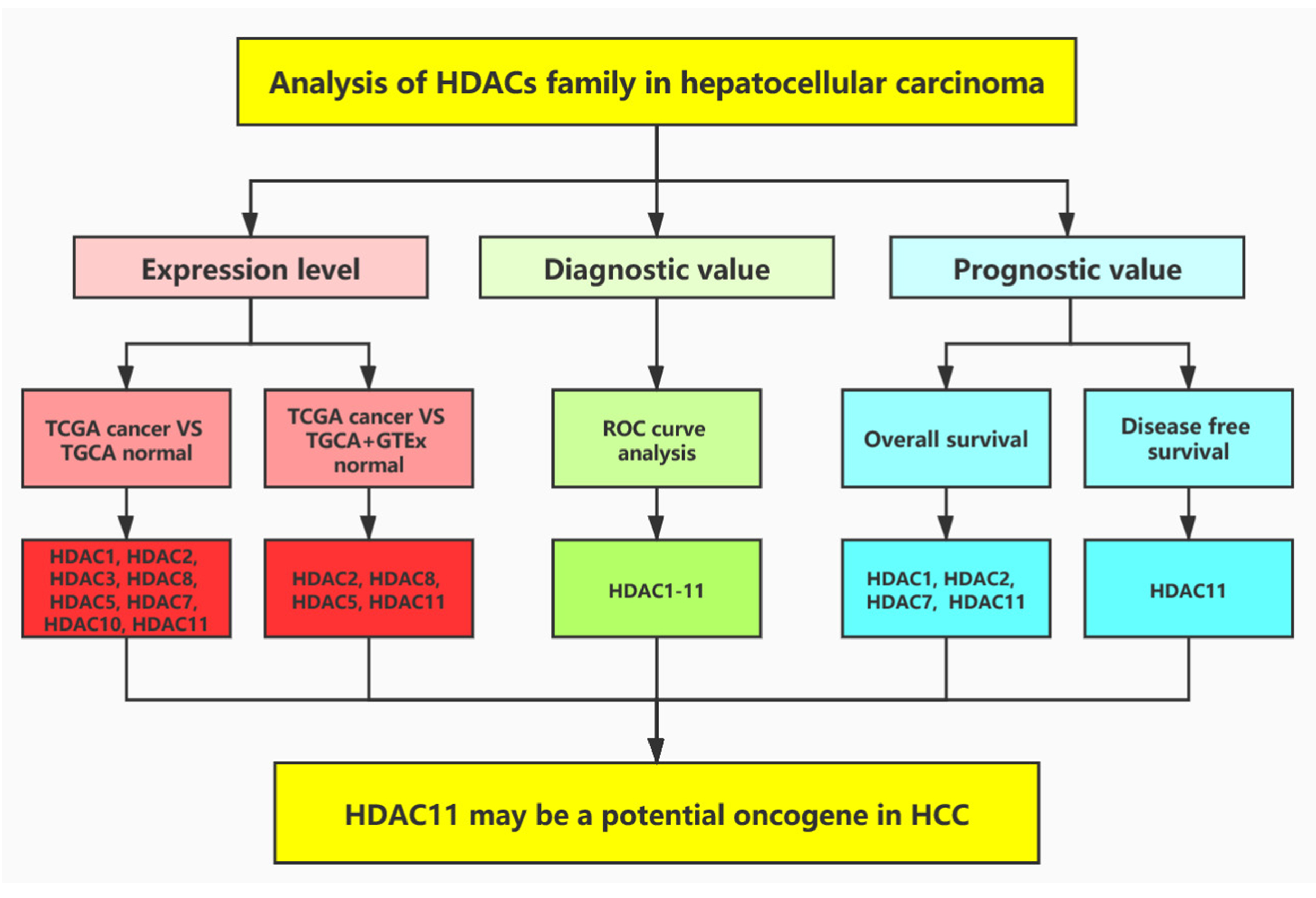

Supplement: FIGURE S3 — The visual flow-process diagram of bioinformatic analyses in this study. [file Image_3.jpeg]

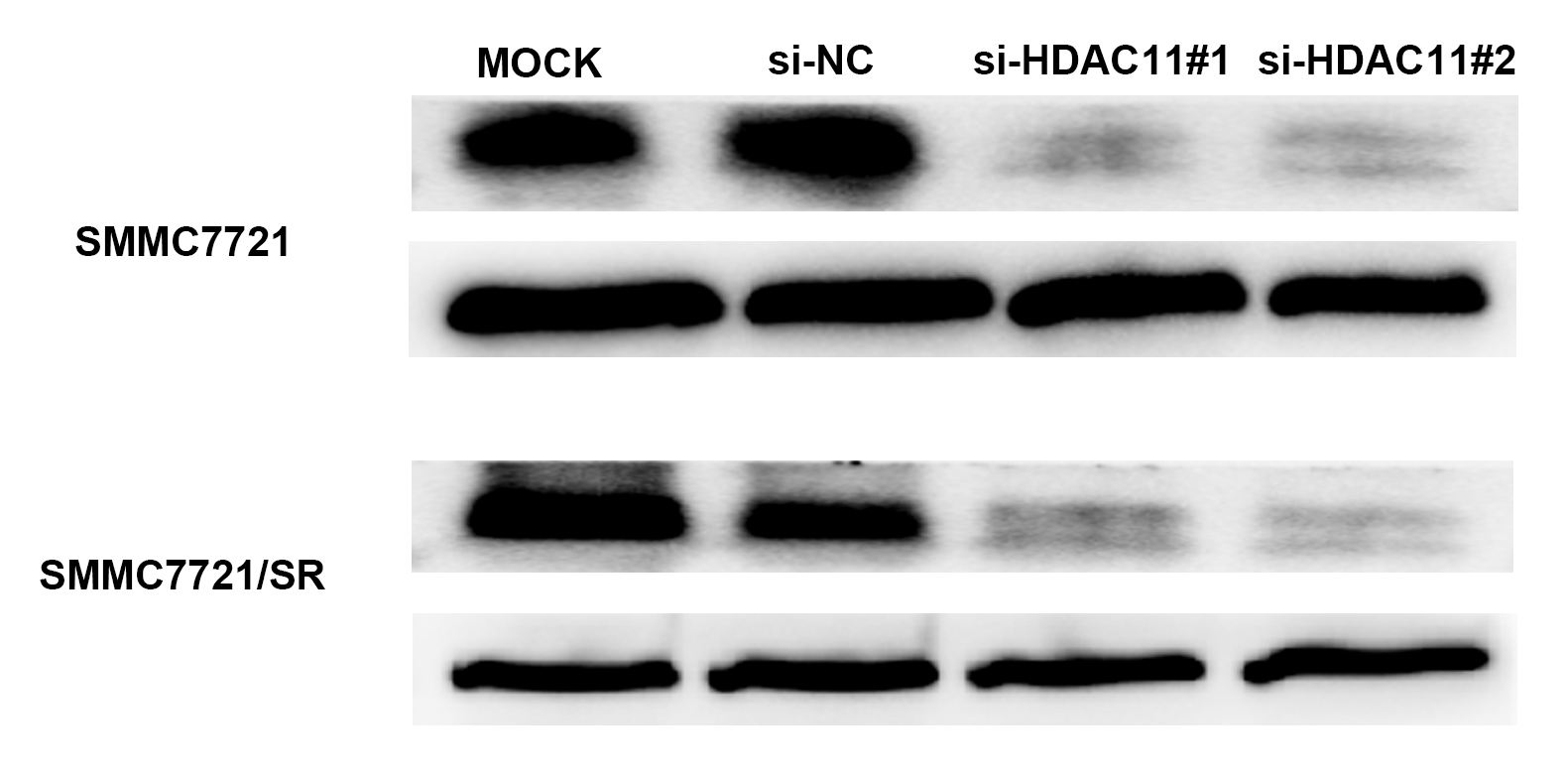

Supplement: FIGURE S4 — The knockdown effect of siRNAs targeting HDAC11 in HCC cells at protein level. [file Image_4.jpeg]

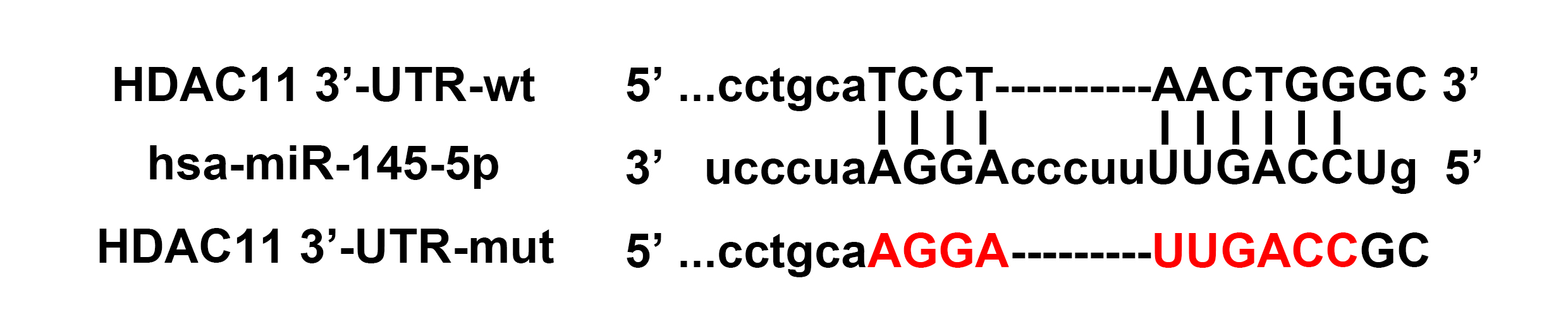

Supplement: FIGURE S5 — The predicted sequences binding regions between wild-type (wt) or mutant (mut) 3′-UTRs of HDAC11 and miR-145-5p. [file Image_5.jpeg]

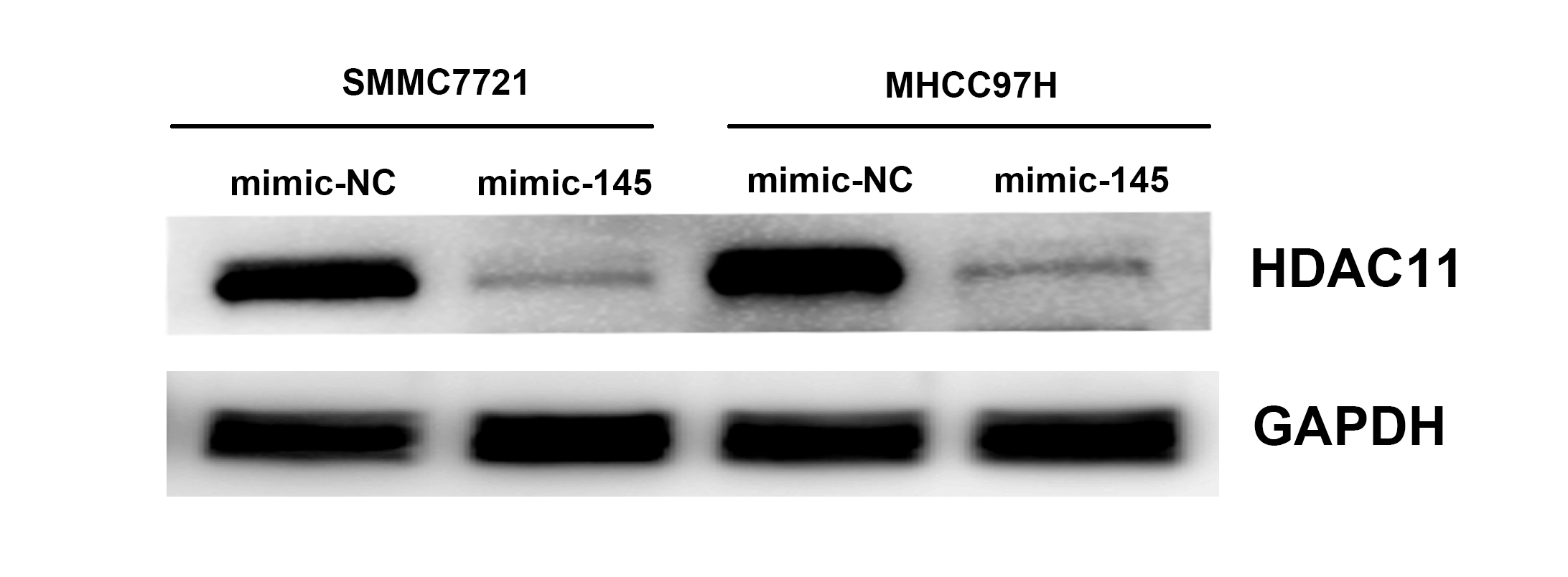

Supplement: FIGURE S6 — Overexpression of miR-145-5p significantly reduced HDAC11 protein level in SMMC7721 and MHCC97H cells. [file Image_6.jpeg]

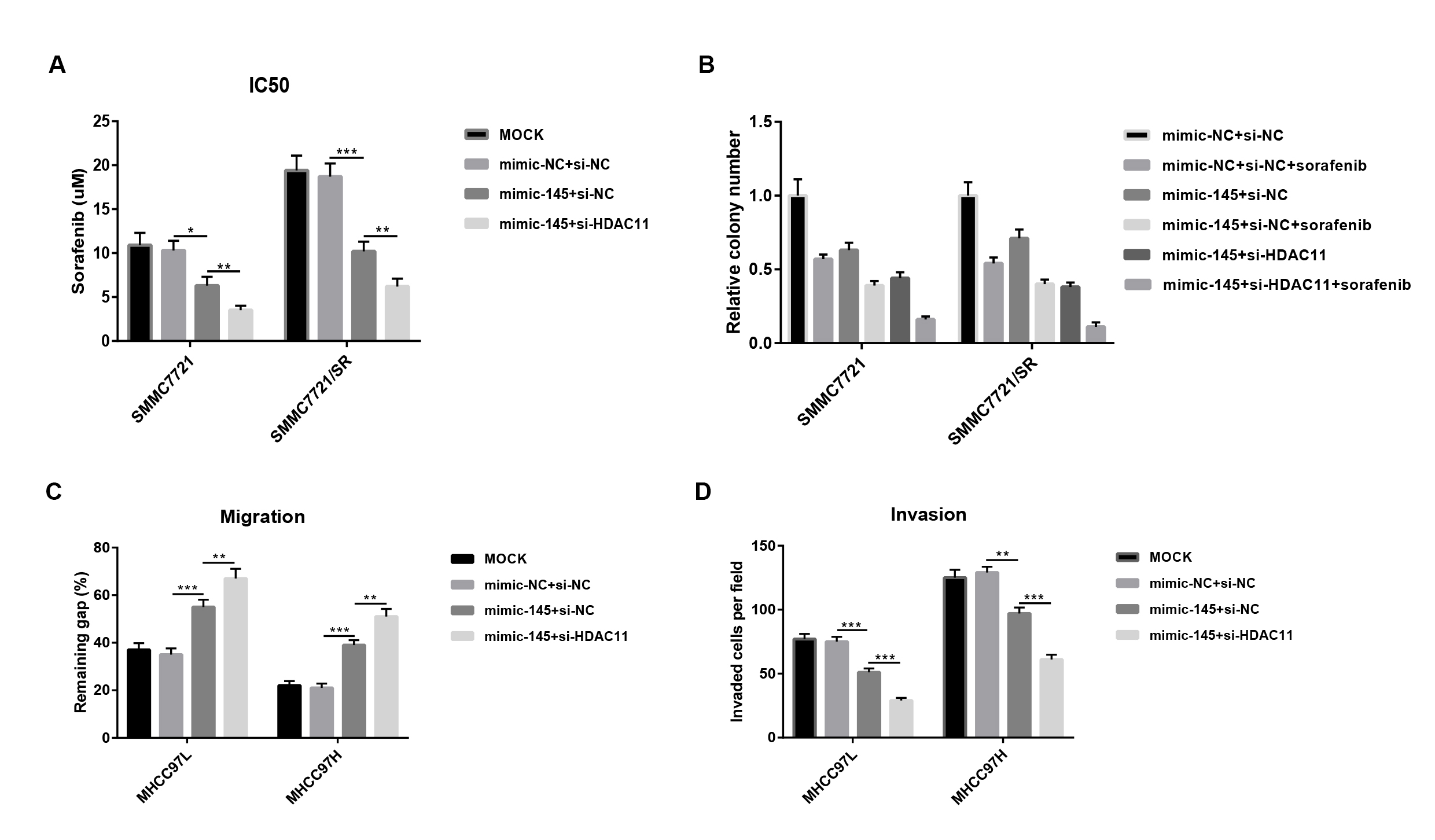

Supplement: FIGURE S7 — The roles of miR-145-5p/HDAC11 axis in sorafenib resistance and metastasis of HCC cells. (A) miR-145-5p overexpression decreased HCC cells resistance to sorafenib, and this effect can be enhanced after knockdown of HDAC11. (B) miR-145-5p overexpression suppressed HCC cell colony formation, and this effect can be enhanced after knockdown of HDAC11. (C) miR-145-5p overexpression inhibited HCC cell migration, and this effect can be enhanced after knockdown of HDAC11. 48 h after wounding. (D) miR-145-5p overexpression suppressed HCC cell invasion, and this effect can be enhanced after knockdown of HDAC11. ∗P < 0.05; ∗∗P < 0.01; ∗∗∗P < 0.001. [file Image_7.jpeg]
